# The Calmodulin-Binding, Short Linear Motif, NSCaTE Is Conserved in L-Type Channel Ancestors of Vertebrate Cav1.2 and Cav1.3 Channels

**DOI:** 10.1371/journal.pone.0061765

**Published:** 2013-04-23

**Authors:** Valentina Taiakina, Adrienne N. Boone, Julia Fux, Adriano Senatore, Danielle Weber-Adrian, J. Guy Guillemette, J. David Spafford

**Affiliations:** 1 Department of Biology, University of Waterloo, Waterloo, Canada; 2 Department of Chemistry, University of Waterloo, Waterloo, Canada; Virginia Commonwealth University, United States of America

## Abstract

NSCaTE is a short linear motif of (xWxxx(I or L)xxxx), composed of residues with a high helix-forming propensity within a mostly disordered N-terminus that is conserved in L-type calcium channels from protostome invertebrates to humans. NSCaTE is an optional, lower affinity and calcium-sensitive binding site for calmodulin (CaM) which competes for CaM binding with a more ancient, C-terminal IQ domain on L-type channels. CaM bound to N- and C- terminal tails serve as dual detectors to changing intracellular Ca^2+^ concentrations, promoting calcium-dependent inactivation of L-type calcium channels. NSCaTE is absent in some arthropod species, and is also lacking in vertebrate L-type isoforms, Ca_v_1.1 and Ca_v_1.4 channels. The pervasiveness of a methionine just downstream from NSCaTE suggests that L-type channels could generate alternative N-termini lacking NSCaTE through the choice of translational start sites. Long N-terminus with an NSCaTE motif in L-type calcium channel homolog LCa_v_1 from pond snail *Lymnaea stagnalis* has a faster calcium-dependent inactivation than a shortened N-termini lacking NSCaTE. NSCaTE effects are present in low concentrations of internal buffer (0.5 mM EGTA), but disappears in high buffer conditions (10 mM EGTA). Snail and mammalian NSCaTE have an alpha-helical propensity upon binding Ca^2+^-CaM and can saturate both CaM N-terminal and C-terminal domains in the absence of a competing IQ motif. NSCaTE evolved in ancestors of the first animals with internal organs for promoting a more rapid, calcium-sensitive inactivation of L-type channels.

## Introduction

Changes in intracellular Ca^2+^ concentrations induce conformational shifts in the ubiquitous Ca^2+^ sensor protein, calmodulin (CaM) [1]. CaM is able to bind up to 300 different known target proteins to date, altering cellular functions [2]. Calmodulin has a particularly unique relationship with calcium-permeant ion channels such as InsP3 receptors [3], ryanodine receptors [4], transient receptor potential channels [5], and high voltage-activated calcium channels [6]. Calcium permeant channels self-regulate their own channel gating when calcium increases are sensed by the CaM sensor located at their intracellular surface, altering their refractoriness or inactivation and preventing excessive calcium entry, or in some cases, facilitate the current [6], [7].

CaM binds to the IQ motifs of C-terminal tails of L-type calcium channels in a parallel orientation with the flexible CaM linker separating its two globular domains (N- and C- terminal domains with doublet pairs of heterogenous, Ca^2+^ -binding EF hands) [8], [9]. The CaM N-terminal domain has lower Ca^2+^ affinity and is sensitive to high intracellular buffering of Ca^2+^, while the C-terminal domain has higher affinity to Ca^2+^ and is buffer resistant [10]. L-type channel homologs from single cell protozoans [11]–[13] and mammalian tissues [6], have calcium-dependent inactivation regulated by a CaM-like protein and shared a conserved C-terminal IQ motif. A second CaM binding motif, NSCaTE in the N-terminus was more recently identified for vertebrate Cav1.2 and Cav1.3 channels [14]. We mined available genomes to determine that NSCaTE appears in L-type calcium channels of all coelomate animals. NSCaTE is a short linear motif composed of residues with high helix-forming propensities, with a key tryptophan (W) residue separated from an isoleucine or leucine (I/L) residue (x-W-xxx (I or L)-xxxx). Binding of NSCaTE to CaM is dependent on rises of intracellular calcium that is sensitive to high intracellular buffering with 10 mM EGTA. A conserved methionine downstream of NSCaTE suggests that there is an alternative translation site for L-type calcium channels for generating L-type channels with and without NSCaTE. The consequence of an optional NSCaTE motif is a much faster calcium-dependent inactivation (CDI) than that contributed by the more ancient IQ motif of L-type calcium channels.

## Results

### L-type Channels have a Conserved Calmodulin-binding IQ Motif Even in Single-celled Paramecium

**Figure 1:**
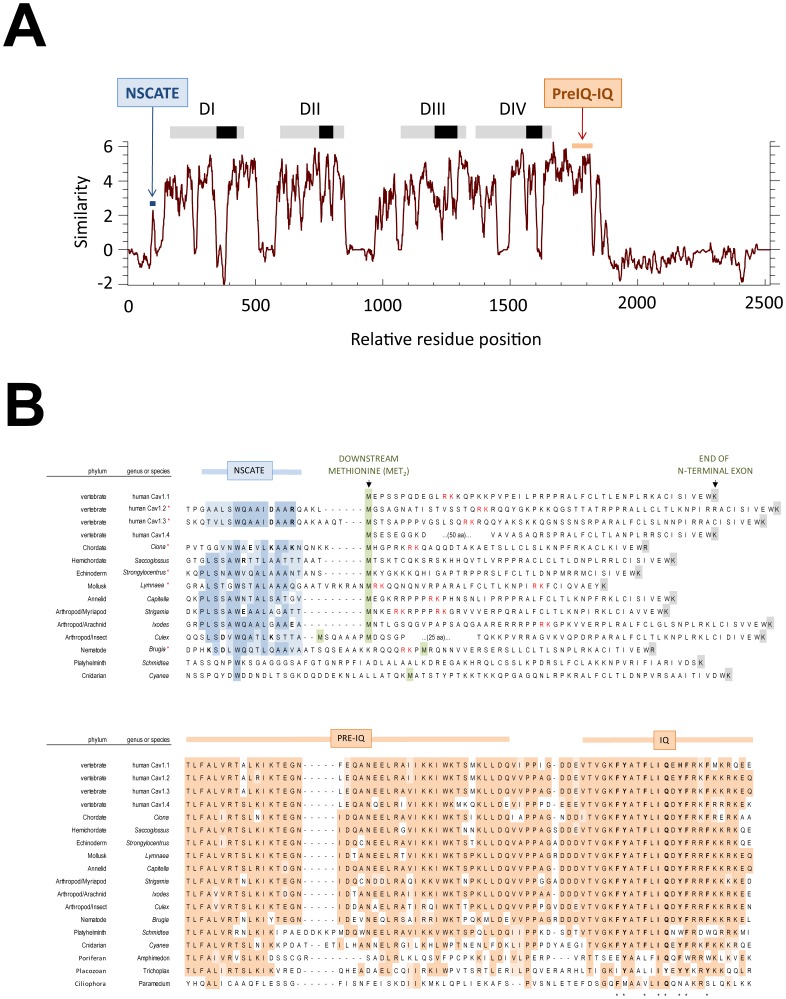
Amino acid sequence conservation of calmodulin binding, amino terminal (NSCaTE) and carboxyl terminal (Pre-IQ/IQ) motifs in L-type Cav1 channels. (A) Running window of amino acid similarity of aligned Cav1.2, Cav1.3 and four representative invertebrate L-type channels (red asterisk in B). DI, DII, DIII, DIV are the location of the four major domains, each domain consisting of six transmembrane helices. (B) Multiple alignment of N-terminal sequences illustrating the conservation of NSCaTE and a downstream methionine (Met_2_) in L-type channels of coelomate animals. (C) C-terminal sequence alignments illustrating the nearly invariant Pre-IQ/IQ motifs in coelomate animals, with a recognizable “IQ” even in single-celled, *Paramecium*.

Voltage-gated calcium channels (Ca_v_ channels) are members of a superfamily of 4×6TM cation channels (4 **D**omains consisting of **6** **T**rans**M**embrane segments) which includes voltage-gated sodium channels and a poorly defined orphan gene NALCN [15], [16]. N- and C-termini flanking the four homologous domains (DI to DIV), and the cytoplasmic linker separating the domains of 4×6TM channels are more variable than the transmembrane domains, when the running average similarity across L-type calcium channels from invertebrate to vertebrate isoforms are compared (**Figure 1A**). Data mining of genomic sequences reveal that all non-vertebrate animals retain a single L-type calcium channel homolog, except for flatworms (platyhelminths) which have two homologs. The mostly single invertebrate L-type channel gene diversified into four vertebrate homologs, Ca_v_1.1 to Ca_v_1.4. (**Figure S1**). Calmodulin binds in a parallel orientation to a canonical C-terminal IQ (isoleucine-glutamine) motif that critically involves six aromatic residue contacts (* residues, in **Figure 1C**) in the crystalized IQ peptide bound to calmodulin [8], [9], and to a secondarily important, upstream Pre-IQ region [17], [18]. Even basal L-type channels in the single-celled ciliates *Paramecium* have an extended C-terminus that includes a conserved IQ motif (**Figure 1C** and **Figure S1**). There are almost identical amino acids for L-type channels from the start of C-terminal cytoplasmic region at the end of the Domain IV through to the IQ motif (**Figure 1A**) in metazoans starting with cnidarians (**Figure 1C** and **Figure S1**). A conserved IQ motif for calmodulin binding is consistent with the observed calcium-dependent inactivation (CDI) of an expressed cnidarian L-type channel homolog [19], and L-type calcium currents in single-celled *Paramecium* that influence swimming and turning behavior [11], [13]. All vertebrate L-type channels possess a CaM binding IQ domain, with calcium dependent inactivation that ranges from minor to very robust, from Ca_v_1.4, to Ca_v_1.1 to Ca_v_1.2 and Ca_v_1.3 channels, respectively [6].

### An N-terminal NSCaTE Motif and Downstream Methionine (Met_2_) is Conserved in L-type Channels from Protostome Invertebrates to Humans

**Figure 2:**
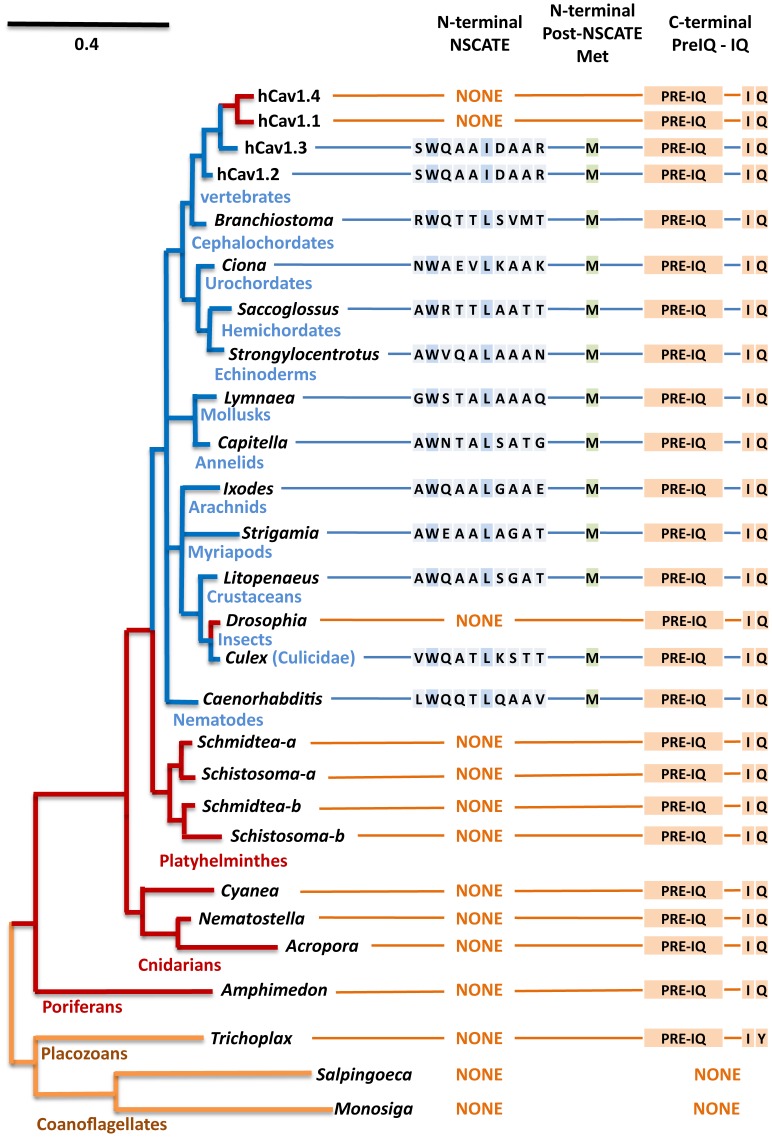
Gene tree of L-type calcium channels illustrating the evolution of calmodulin binding, amino terminal (NSCaTE) and carboxyl terminal (Pre-IQ/IQ) motifs in L-type Cav1 channels. All animal phyla including single cell protozoans (such as *Paramecium*) and simple multicellular organisms (placozoans, sponge) have an IQ motif. A N-terminal NSCaTE (xW*xxx*(IorL)*xxxx*) motif evolved in common invertebrate ancestors (coelomates) and retained in all major phylogenetic groups. While the Pre-IQ and IQ motifs are featured in all L-type channels of metazoans, the NSCaTE motif is missing in some Arthropod species, including many insects, suggesting that NSCaTE is not an essential feature of L-type channels. NSCaTE was lost in Ca_v_1.1 and Ca_v_1.4 after the speciation of L-type channels to four gene isoforms in vertebrates. Almost every NSCaTE containing L-type channel has a downstream methionine (Met_2_) from the start codon (Met_1_) which could serve as an alternative translational start site for inclusion (Met_1_) or exclusion (Met_2_) of NSCaTE in L-type channels.

The N-termini of L-type channels are highly-variable, and predicted to be mostly disordered sequence (DISOPRED software prediction, **Figure S3**) that ends at a downstream, often U12-type, AT-AC intron splice junction (**Figure 1B** and **Figure S2**), shared between Ca_v_ and Na_v_ channels. All animal phyla starting with protostomes (nematodes, annelids, mollusks, arthropods) possess a conserved NSCaTE motif, which went previously unreported in the discovery of NSCaTE in vertebrates because of the high variability in the N-terminal sequences of L-type channels [14]. NSCaTE is a canonical short Linear Motif (SLiM), with a short (<12 aa) contiguous amino acid stretch of amino acids (x-**W-**xxx-(**I or L**)-xxxx) that have a high propensity to undergo structural change upon binding of a ligand, and possesses a few critical hotspot binding residues, always Trp (**W**) and invariantly, four residues downstream from Trp, an Iso (**I**) in vertebrates, or Leu (**L**) in invertebrates (**Figure 1B** and **Figure S2**). NSCaTE is absent in some arthropod species, notably all insects, except for the mosquito family (Culcidae), absent in all vertebrate Ca_v_1.1 and Ca_v_1.4 channels, and lost in some species of arachnids and crustaceans (**Figure 2**). Another striking feature of NSCaTE-containing N-termini is the almost universal presence of a second methionine Met_2_ (usually 0 to 10 amino acids downstream from NSCaTE outside of nematodes) (**Figure 2**). We expect generation of a long form NSCaTE-containing L-type channel translated with upstream Met_1_ and an alternative short isoform, L-type channel lacking NSCaTE translated from Met_2_ downstream of the NSCaTE sequence (**Figure 1B** and **Figure S2**). The absence of a splice junction in the long N-termini of most L-type channels (exceptions being vertebrate Cav1.2 and Cav1.3 channels and some nematodes), suggest that L-type channels likely utilize alternative translational start sites to generate long and short N-termini to retain or exclude the NSCaTE motif, rather than utilize an exon-skipping mechanism.

### No Notable Differences in the mRNA Levels for NSCaTE (LCav_1_-Met_1_) from those Lacking NSCaTE (LCav_1_-Met_2_)

**Figure 3:**
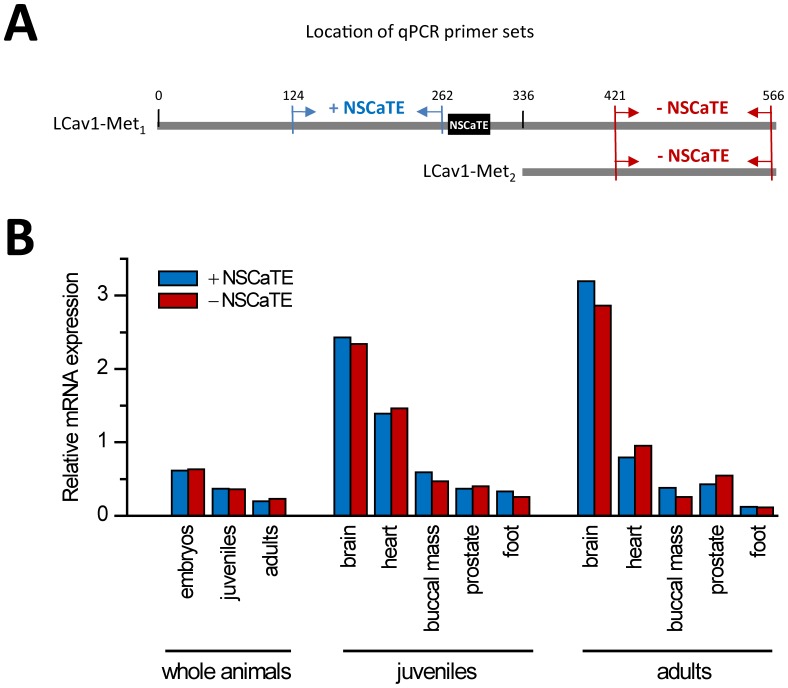
No difference in levels between snail LCa_v_1 (L-type) channel mRNA transcripts containing NSCaTE (LCa_v_1-Met_1_) and those lacking NSCaTE (LCa_v_1-Met_2_). A) Illustration of the location of primer sets for qPCR amplification of snail mRNA transcripts containing or lacking NSCaTE in LCa_v_1 channels. Numbers refer to the position of the DNA sequence from LCav1-Met_1_ translational start site. B) Graph illustrates the lack of significant difference in mRNA expression levels (relative to HPRT control values, scaled such that the sum of all signal values across tissues are equal) in whole animals or in specific tissues of snails containing or lacking the LCav1_NSCaTE_ sequence. Degree of correlation with adjusted R^2^ value = 0.95.

We rule out the possible differential regulation of mRNA transcription of LCav_1_-Met_1_ versus LCav_1_-Met_2_ (**Figure 3**). We observed no transcriptional differences when we quantified the amplification of reverse-transcribed mRNA isolated from whole snails at different developmental stages (100% embryos, juveniles and adults) and different tissues (brain, heart, buccal mass, prostate, foot) of juvenile and adult animals (**Figure 3B**) using primer sets for quantitative RT-PCR (qPCR) spanning regions upstream (+NSCaTE) or downstream (-NSCaTE) of NSCaTE (**Figure 3A**).

### No Obvious Biophysical Differences in LCav1 Channels Containing NSCaTE (LCav_1_-Met_1_) from those Lacking NSCaTE (LCav_1_-Met_2_)

**Figure 4:**
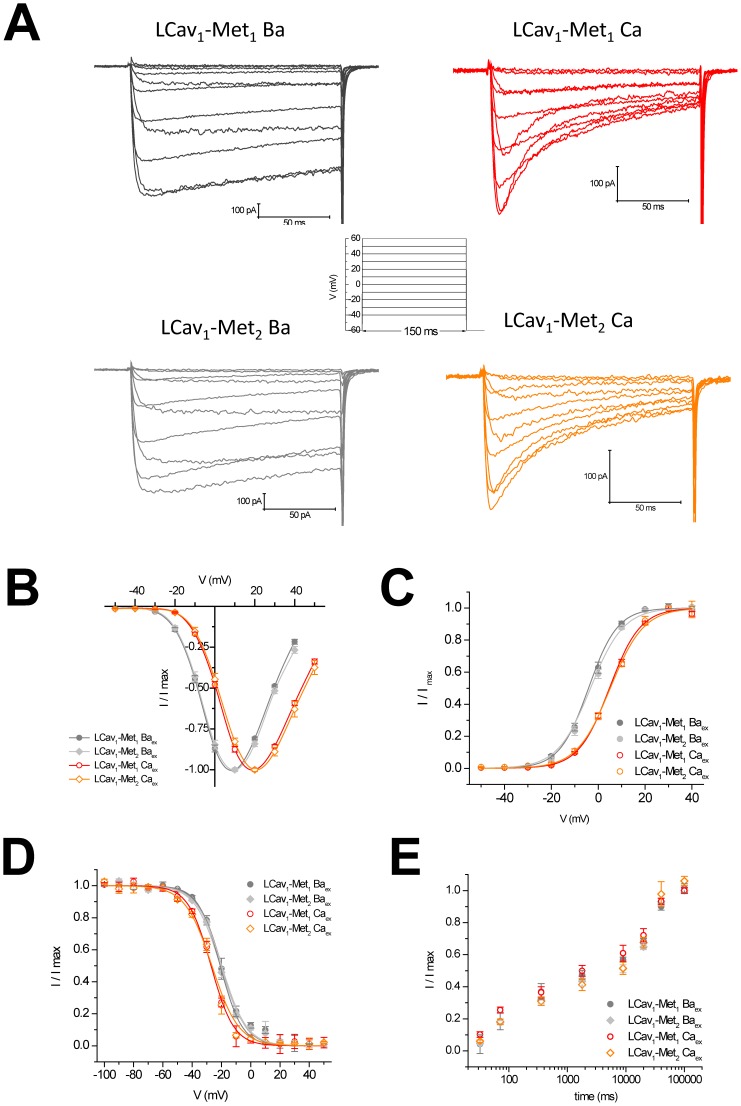
No major differences in biophysical properties between snail LCa_v_1 channel containing a full-length N-terminus with NSCaTE translated from upstream methionine Met_1_ or truncated N-terminus missing NSCaTE generated from the downstream methionine Met_2_. LCa_v_1-Met_1_ and LCa_v_2-Met_2_ were transfected in HEK-293T cells alongside mammalian α_2_δ_1_ and β1b accessory subunits and recorded in 10 mM Barium (Ba) or Calcium (Ca) containing extracellular solution using patch clamp electrophysiology. Intracellular solution contained 9 mM EGTA. (A) Representative current traces generated from voltage steps (−40 mV to 60 mV in 10 mV steps) from a holding potential of −60 mV) illustrating the typical buffer resistant (9 mM EGTA) calcium-dependent inactivation when calcium is the charge carrier, leaving residual voltage-dependent inactivation when barium replaces calcium in the extracellular solution. (B) Normalized current-voltage relationships (n = 10), transformed and Boltzmann-fitted as activation curves in (C). (D) Steady-state availability curves (Ba_ex_: n = 10, Ca_ex_: n = 6), generated by measuring the fraction of maximal current generated after a 10 s sustained prepulse voltage from −100 to +50 mV in 10 mV steps). (E) Time of recovery from inactivation (n = 4) measured as the fraction of maximal current recovery after time delays, plotted on a log scale.

We test the functional relevance of alternative start sites with the singleton, L-type calcium channel gene (LCav1), isolated from the pond snail *Lymnaea stagnalis* [20], [21]. The snail LCav1 channel is more distantly related in overall protein sequence similarity to vertebrate isoforms, Cav1.1 to Cav1.4 isoforms, diverging in structure likely from the time of the likely branching of the lineage of gastropod snails, ∼ ½ billion years ago [20]. We observed no apparent differences in biophysical properties of snail isoforms of full length N-terminus containing NSCaTE, *LCav_1_-Met_1_* when comparing to the shorter N-terminus lacking NSCaTE *LCav_1_-Met_2_* expressed and recorded in HEK-293T cells using whole-cell patch clamp electrophysiology in standard intracellular solutions containing 9 mM EGTA (**Figure 4** and **Table 1**). Ensemble of barium currents generated from voltage-steps from a resting membrane potential (−60 mV) especially to a peak current size, reveal the typical slower inactivation decay rate compared to the calcium current decay rate over the 150 ms depolarizing pulse, indicative of a faster calcium-dependent inactivation process, in addition to the voltage-dependent inactivation decay component revealed when barium is the charge carrier (sample traces in **Figure 4A**). LCa_v_1 barium currents were typically 2 fold larger than equivalent calcium currents, which is a property shared for all snail calcium channels, including LCa_v_2 (Non-L-type) [22], [23] and LCa_v_3 (T-type) [24]. Barium and calcium currents were shifted relative to each other in their voltage at which they reached a peak current (**Figure 4B**), that reflected a voltage shift in the activation conductance (**Figure 4C**). There were also differences in steady-state availability curves comparing barium and calcium as the charge carrier (**Figure 4D**). Nonetheless, no apparent biophysical changes were imparted by the presence of the NSCaTE containing peptide spanning Met_1_ and Met_2_ of the LCa_v_1 channel (**Figure 4** and **Table 1**).

**Table 1:** Summary of biophysical parameters of LCav3 channel variants containing exons 8b and 25c expressed in HEK-293T cells, with one-way analysis of variance to assess statistical significance.

			[Ba_ex_] = 10 mM						[Ca_ex_] = 10 mM									
			LCav_1_Met_1_			LCav_1_Met_2_			LCav_1_Met_1_			LCav_1_Met_2_			[Ba_ex_]	[Ca_ex_]	[Ba_ex_] vs [Ca_ex_]	
			n	mean	SEM	n	mean	SEM	n	mean	SEM	n	mean	SEM	Met_1_ vs Met_2_		Met_1_	Met_1_
**[EGTA]_in_ = 9 mM EGTA**																		
**Activation:**																		
V1/2 (mV)			10	3.31	0.28	10	−3.13	0.32	10	5.17	0.26	10	5.12	0.36	n.s.	n.s.	***	***
slope			10	5.90	0.33	10	5.92	0.28	10	6.49	0.20	10	6.83	0.32	n.s.	n.s.	***	***
**Inactivation:**																		
V1/2 (mV)			10	−19.78	1.12	10	−20.69	1.18	6	×25.69	1.14	6	−25.32	1.10	n.s.	n.s.	***	***
slope			10	8.14	0.99	10	8.67	1.04	6	7.74	1.03	6	6.61	0.96	n.s.	n.s.	***	***
**Inactivation recovery:**																		
% recovery at 1 sec			4	41.88%	2.56%	4	40.87%	3.30%	4	46.87%	3.31%	4	39.82%	2.33%	n.s.	*	*	n.s.
**I_max_ amplitude:**																		
Ba:Ca size ratio			10	2.02	0.19	6	1.91	0.12							n.s.			
**Inactivation kinetics:**																		
R_300_			5	0.91	0.023	5	0.89	0.038	5	0.32	0.013	5	0.31	0.034	n.s.	n.s.	***	***
**[EGTA]_in_ = 0.5 mM EGTA**																		
**Inactivation kinetics:**																		
R_300_			5	0.85	0.012	5	0.86	0.020	5	0.18	0.023	5	0.37	0.034	n.s.	***	***	***
n.s. not significant;																		
*p<0.05;																		
**p<0.005;																		
***p<0.001.																		

### A buffer-sensitive Calcium-dependent Inactivation is Uncovered with NSCaTE Containing Channels (LCav_1_-Met_1_) in Low 0.5 mM EGTA Intracellular Recording Buffer

**Figure 5:**
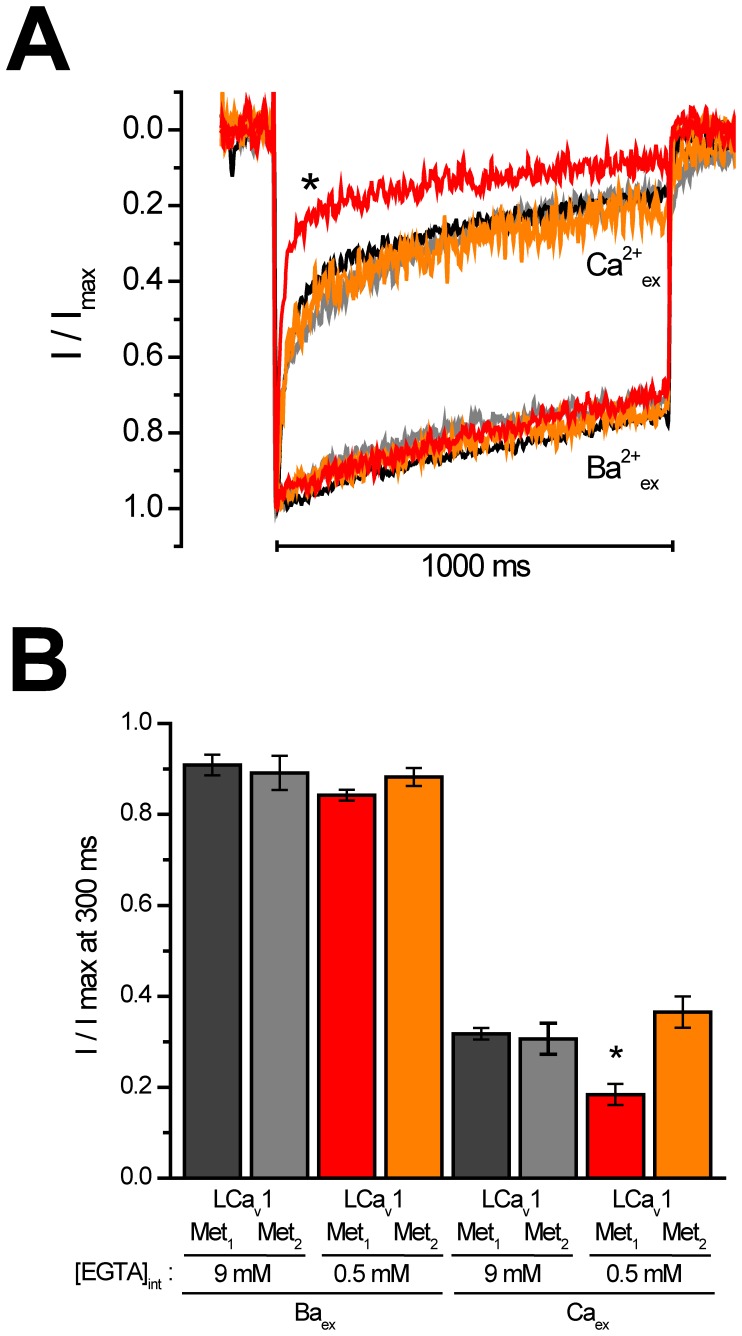
Full-length LCa_v_1-Met_1_ channels containing NSCaTE have a buffer-sensitive form of calcium dependent inactivation not found in truncated LCa_v_1-Met_2_ channels lacking NSCaTE. LCa_v_1-Met_1_ and LCa_v_2-Met_2_ were transfected in HEK-293T cells alongside mammalian α_2_δ_1_ and β2a accessory subunits and recorded using patch clamp electrophysiology. (A) Overlapping sample traces illustrating the ultra-fast calcium-dependent inactivation (red trace) of LCav_1_-Met_1_ channel currents in low (0.5 mM) EGTA buffering conditions. (B) Graph of fraction of peak current size at 300 ms time point of inactivation decay (R300). * represents a statistically significantly smaller R300 value (p<0.001, ANOVA) for LCav_1_-Met_1_ calcium currents (red trace), reflecting the buffer-sensitive calcium dependent inactivation uniquely in LCav_1_ channels with a full N-terminus, containing NSCaTE.

A function for NSCaTE is revealed when a more physiologically-relevant low buffering conditions (0.5 mM of calcium-chelator, EGTA) replaces the standard high buffering conditions of 9 mM of EGTA. Large calcium and barium currents were generated from voltage-steps to +10 mV from a holding potential of −100 mV over a 1 s depolarizing pulse. Barium and calcium currents generated from multiple cells (n = 5) were assessed for intracellular conditions of high buffering (9 mM EGTA) and low buffering (0.5 mM EGTA)**.** Only the LCav1-Met_1_ channel currents in low buffering conditions reveal a much more rapid calcium dependent inactivation and this was not evident in LCav1-Met_2_ channel currents lacking NSCaTE (**Figure 5** **).** A similar loss of calcium dependent inactivation in high, but not low buffering condition is featured of synaptic Cav2 channels [6], [25]. The ultra-fast, and significantly faster calcium dependent inactivation imparted by the NSCaTE containing N-terminus, is evident when individual current traces are overlapped (**Figure 5A**), and illustrated as the fraction of residual current at the 300 ms time point of inactivation decay (R300 value, mean +/− SEM (**Figure 5B** **and** **Table 1**).

### Snail NSCaTE Assumes an Alpha-helix Upon Ca^2+^-CaM Binding

**Figure 6:**
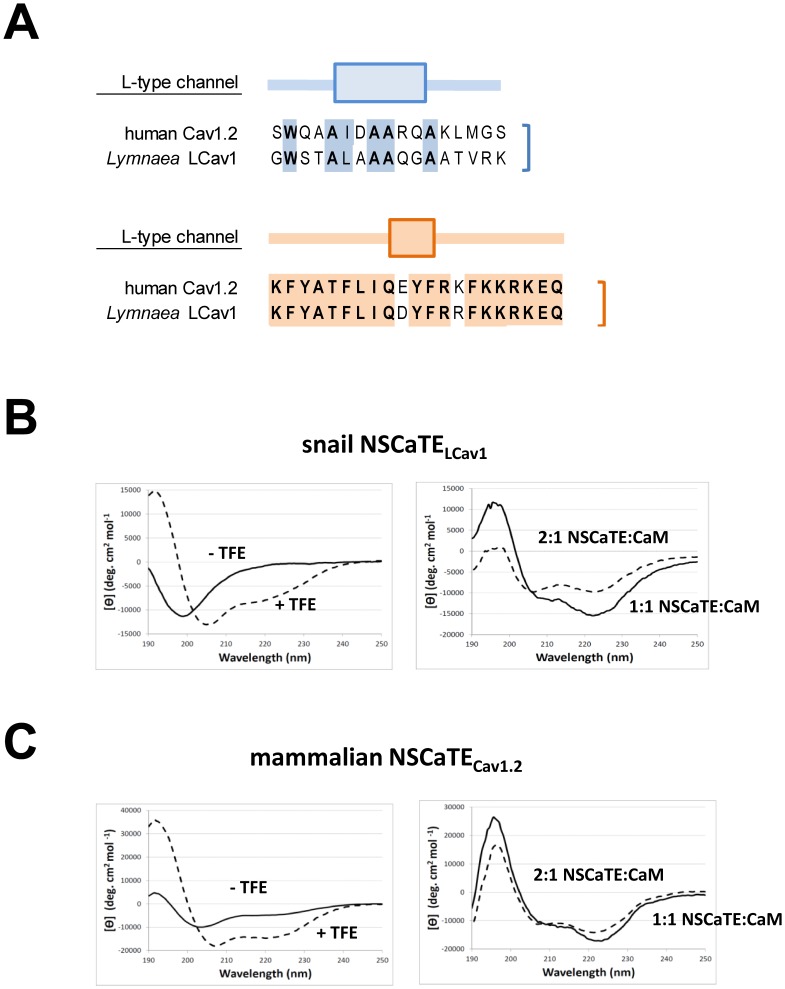
Snail NSCaTE_LCav1_ and mammalian NSCaTE_Cav1.2_ peptides assume an alpha-helix upon binding calmodulin (CaM). (A) 17 mer NSCaTE and 21 mer IQ peptide sequences are used in circular dichroism (Figures 6) and Gel Shift Mobility Assays (Figures 7). (B,C) Differential spectra of NSCaTE peptide indicate a helical transformation with snail and mammalian NSCaTE upon addition of the helix stabilizing agent, trifluoroethanol (TFE: dashed line; no TFE: solid line, Figure 6B) or upon addition to CaM (solid line = 1∶1 CaM:peptide, dashed line = 1∶2 CaM:peptide, Figure 6C). Each trace is obtained by subtracting the 10 µM CaM-alone trace from the corresponding NSCaTE+CaM spectrum. Y axis units are mean residue ellipticity or (θ), (difference in spectra corrected for total protein/peptide concentration).

Snail NSCaTE contained in the full N-terminus of LCav_1_-Met_1_ is expected to compete with the canonical IQ motif to associate with calcium-calmodulin (Ca^2+^-CaM) and to generate a buffer-sensitive component to the current and a more rapid form of calcium dependent inactivation for L-type channels. Binding of CaM to target peptides is typically associated with conformational changes, with a marked increase in alpha-helical content [26]. Indeed, this is what we observe with our spectropolarimetry results (illustrated in **Figure 6B** **,** **Figure 6C**).

21mer peptides for snail IQ_LCav1_ and human IQ_LCav1.2_ are 100% similar in sequence to one another, but are not identical (90.5%) (**Figure 6A**). In comparison snail NSCaTE_LCav1_ is remotely recognizable over a 17 mer peptide sequence with only 29.4% of shared amino acids to mammalian NSCaTE_Cav1.2_ (**Figure 6A**). Outside of the conserved W and I/L positions in snail and mammalian NSCaTE, the most common amino acid are four shared alanines (A), which have a high alpha-helical propensity (**Figure 6A**). The region surrounding almost every putative NSCaTE sequence forms a predicted alpha helix (PSIPRED3.3, UCL-CS Bioinformatics) [27], contained in a highly disordered/unstructured N-terminus (DISOPRED2, UCL-CS Bioinformatics) [28] in deuterostomes and annelids (**Figure S3**) and mollusks, arthropods and nematodes (**Figure S4**) (**Table S1**).

The expected alpha-helical transformation of NSCaTE peptides is evident from the absorption spectra at 208 and 222 nm using plane polarized light [29]. Snail NSCaTE_LCav1_ peptide goes from an unstructured conformation to an alpha-helix upon addition of helical stabilizing agent, tetrafluoroethanol (TFE) or upon titration of CaM to snail NSCaTE_LCav1_ at a 1∶1 ratio of NSCaTE peptide to CaM (**solid to dashed lines in** **Figure 6B** **)**. The conformational change associated with NSCaTE_LCav1_ binding to CaM was observed upon addition of NSCaTE_LCav1_ peptide into a fixed concentration of CaM. Resultant spectra were subtracted, smoothed and corrected for the total protein/peptide concentration. Interestingly, the maximum increase in alpha-helicity for snail NSCaTE_LCav1_ decreases slightly upon further addition of NSCaTE_LCav1_ from a 1∶1 to a 2∶1 ratio to NSCaTE peptide:CaM (**Figure 6B**). This could be in part due to more unbound (thus unstructured) peptide in solution, or a less constrained alpha-helical nature of the secondary binding site.

Mammalian NSCaTE_Cav1.2_ peptide is modestly structured as a helix in solution compared to the completely unstructured snail NSCaTE_LCav1_ in solution. Mammalian NSCaTE_Cav1.2_ peptide increases in relative alpha-helicity upon addition of TFE or upon titration of CaM to NSCaTE_Cav1.2_ (**solid to dashed lines in** **Figure 6C** **)** to a maximum alpha-helicity that is much greater than that achieved by snail NSCaTE_LCav1_ (**compare** **Figures 6B** **and** **Figure 6C**). Both snail and mammalian NSCaTE peptides undergo a significant structural change upon binding CaM, with an apparent difference being in a modest alpha-helical conformation of the unbound mammalian NSCaTE_Cav1.2_ peptide. A similar helical structure of the native mammalian NSCaTE_Cav1.2_ peptide in solution was reported by Liu and Vogel, 2012 [30].

### Snail NSCaTE can Associate with CaM Pre-bound to the IQ Motif

**Figure 7:**
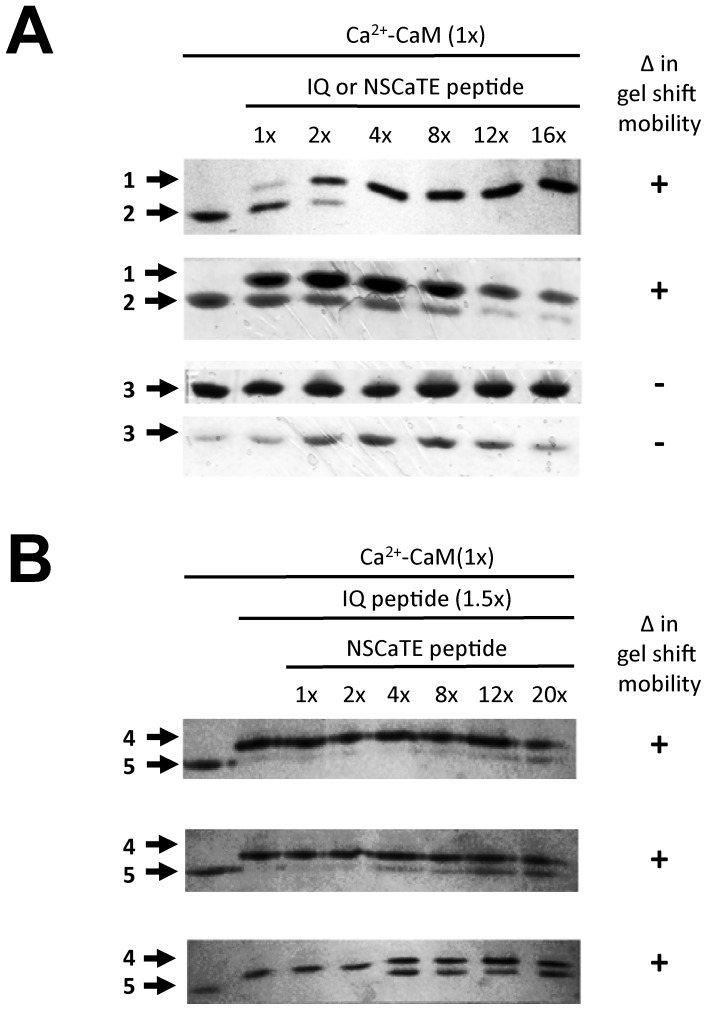
Gel Mobility Shift Assays illustrate that snail NSCaTE of LCav1 and mammalian NSCaTE of Cav1.2 can displace calcium-calmodulin (Ca^2+^-CaM) prebound to either snail IQ or mammalian IQ motifs. Gel shift mobility assays of CaM and individual peptides (A) or CaM pre-bound to an IQ peptide competing with increasing amounts of NSCaTE (B). Each lane contains 300pmol wild-type Ca^2+^-CaM; first lane is a CaM-only control for reference (Positions #2,#3,#5). (A) Each subsequent lane has increasing ratios of C-terminal (IQ) and/or N-terminal NSCaTE peptides at the ratios indicated. The changing conformation of CaM by IQ peptide causes a mobility shift of the CaM band (from Positions #2 → #1, top panels). Neither snail NSCaTE_LCav1_ or mammalian NSCaTE_Cav1.2_ changed CaM mobility alone, at any ratio (Position #3, bottom panels). (B) First lane is again Ca^2+^-CaM-only control (Position #5). Second lane is Ca^2+^-CaM with 1.5×IQ peptide alone control (for the maximum shift reference, Position #4). In subsequent lanes, increasing the amount of added NSCaTE peptide eventually reversed the slow mobility of CaM-IQ peptide back to the faster mobility of CaM without IQ peptide (Position #4 → #5), but not at a 100% effectiveness.

The conformation of CaM strongly affects its rate of migration in poly-acrylamide gels. We used this property to analyze the interaction of CaM with the competing L-type channel termini: the N-terminal NSCaTE and C-terminal IQ motif binding peptides. Increasing concentrations of snail or mammalian C-terminal IQ peptides promotes a slight decrease in Ca^2+^-CaM mobility on gels, while no Ca^2+^-CaM mobility change was evident with addition of snail or mammalian NSCaTE peptide (**Figure 7A**). These results are consistent with CaM binding to NSCaTE peptides in a more compact conformation than the IQ peptides. When CaM is pre-bound to the IQ peptide first, and an increasing amount of NSCaTE peptide is added, the addition of excess NSCaTE peptide causes a reversal of the mobility shift back to a more compact, faster migrating CaM species, although never at a 100% effectiveness (even at a 10 fold or greater excess of NSCaTE peptide), due to its lower affinity for CaM (**Figure 7B**). The most likely explanation is that CaM binds the IQ and NSCaTE peptide at two distinct sites (likely its C and N lobe, respectively), which is further supported by our calorimetry data (see below), and is consistent with a recent study of mammalian NSCaTE [30]. Both snail NSCaTE_LCav1_ and mammalian NSCaTE_Cav1.2,_ were able to displace their respective IQ peptides (**Figure 7B**), despite the low conservation (29.4% identity) between the NSCaTE sequences (**Figure 6A**).

### Snail NSCaTE Binds to CaM in a 2∶1 NSCaTE : CaM Ratio

**Figure 8:**
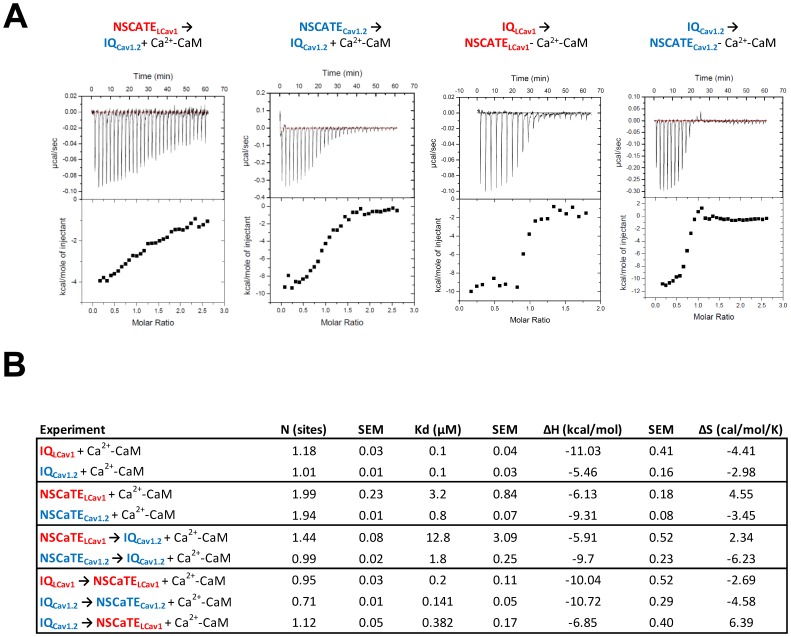
Isothermal Calorimetry (ITC) analysis indicates a 2∶1 stoichiometry of NSCaTE:CaM in the absence of IQ, and a 1∶1 NSCaTE:CaM stoichiometry when CaM is first pre-bound to IQ. (A) Representative raw sample data for several CaM-peptide titrations. (B) Summary Table of ITC data. NSCaTE or IQ peptides were titrated into Ca^2+^-CaM alone or Ca^2+^-CaM pre-bound to a competing peptide at a 1∶1 ratio. All binding reactions exhibited negative enthalpy under the experimental conditions used (exothermic, ΔH <0). Despite the lack of mobility shift of Ca^2+^-CaM (Figure 7), NSCaTE does bind Ca^2+^-CaM alone. LCa_v_1 and Ca_v_1.2 IQ peptides have a higher affinity (Kd = 80 nM and 130 nM, respectively) for Ca^2+^-CaM than do either of the NSCaTE peptides (Kd = 0.83 µM and 3.24 µM for Ca_v_1.2 and LCa_v_1 NSCaTEs). Both mammalian NSCaTE_Cav1.2_ and snail NSCaTE_LCav1_ is able to bind to Ca^2+^-CaM pre-bound to IQ motifs, and both IQ peptides are able to bind to CaM when it is first bound to NSCaTE, which is consistent with the possibility of a NSCaTE-CaM-IQ complex *in vivo*. N values indicate that NSCaTE_LCav1_ and NSCaTE_Cav1.2_ can bind to Ca^2+^-CaM in a 2∶1 stoichiometry, consistent with NSCaTE possibly binding simultaneously to both the N- and C-lobes of CaM, as previously reported [30].

Thermodynamic parameters of the NSCaTE and IQ peptide interactions with Ca^+^-CaM were quantified using isothermal titration calorimetry or ITC (**Figure 8**). MicroCal LLC software was used to perform regression analysis and least-squares fitting of the data using the one-set-of-sites model. The calorimetric data indicate a stronger affinity of CaM for the IQ motif peptides ∼100 nM) and weaker affinity for NSCaTE peptides (≥1 µM) (**Figure 8**). The weaker NSCaTE binding is consistent with published reports [14], [30]–[33]. Despite an apparent lack of mobility change with CaM (**Figure 7A**), the ITC results confirm that both snail and mammalian NSCaTE bind CaM without a prebound IQ motif, although affinities differ, with the snail NSCaTE_LCav1_ being weaker than mammalian NSCaTE_Cav1.2_ (mammalian NSCaTE_Cav1.2 _Kd = ∼0.8 µM and snail NSCaTE_LCav1_ Kd = ∼3.2 µM for) (**Figure 8**). In addition, the snail NSCaTE_LCav1_ appears to have a greater contribution from the entropy of binding to CaM (ΔS = 4.5 cal mol^−1^ K^−1^) than the mammalian NSCaTE_Cav1.2_, whose binding appears to be entropically unfavorable (ΔS = −3.4 cal mol^−1^ K^−1^) at 25°C. This is possibly explained by the higher hydrophobic content of the snail NSCaTE_LCav1_, which would presumably increase the surface area buried during binding, reflecting an increase in the entropy of excluded solvent.

The calorimetric data suggest that both snail and mammalian NSCaTE associate with CaM in the presence or absence of a pre-bound IQ motif. All binding interactions between Ca^2+^-CaM and either or both NSCaTE and IQ motifs were exothermic (negative enthalpy change) (**Figure 8**). The positive entropy change persisted for the snail NSCaTE with all the competition experiments, which suggests that its primary binding conformation with CaM is not affected by the competing IQ peptide (which likely binds at a different location). Negative enthalpy of binding is characteristic of CaM binding to unstructured peptides [34], which is consistent with our spectropolarimetry results. As expected, none of the peptides were able to bind calcium-free calmodulin or apo-CaM, as seen by the lack of binding in the presence of excess EGTA or EDTA (data not shown).

Furthermore, the isothermal titration calorimetry (ITC) results indicate that NSCaTE binds CaM in a 2∶1 ratio, similarly to a recently published work that suggests each lobe-domain of CaM is able to bind the NSCaTE peptide independently [30]. The 2∶1 stoichiometry of NSCaTE binding to CaM is evident for both snail and mammalian NSCaTE (**Figure 8**). It is possible that NSCaTE and IQ motifs are able to both bind CaM simultaneously, in a novel, previously undocumented configuration. This is supported by the fact that stoichiometry of NSCaTE binding to CaM is approximately 1∶1 when CaM is first prebound to the IQ peptide rather than 2∶1 with NSCaTE alone, suggesting that both NSCaTE and IQ peptides may be competing for the high affinity site on CaM. It is also possible that CaM is bridging the two cytoplasmic regions of L-type channels *in vivo* even if such an interaction is weak and/or transient. The incomplete displacement of IQ peptide by excess NSCaTE peptide in our gel shift data (illustrated in **Figure 7**) supports the hypothesis that the IQ peptide forces CaM into a more open conformation, causing any CaM that is bound to the IQ peptide to migrate slowly (regardless of whether that CaM is also bound to an NSCaTE peptide). In contrast, any CaM that is free or bound only to NSCaTE will revert to a faster migrating form of CaM. Large excess of NSCaTE peptide cannot completely displace all of the IQ peptide from CaM (**Figure 7B**), since the affinity of IQ for CaM is greater by 10 fold or more (**Figure 8**). The result is the presence of a slower migrating CaM bound to IQ motif even in excess of NSCaTE peptide (**Figure 7**B).

## Discussion

### Evolution of Calcium-dependent Inactivation

Calcium channels enter a refractory or inactivated state, resistant to channel opening if they are open for an extended period but then turn off as if it has a spring-loaded timer, preventing excessive calcium entry that is toxic to the cellular interior that is normally highly-buffered and kept at exquisitely low levels (100 nM) within cells. Calmodulin (CaM) associated with a C-terminal IQ domain of L-type channels was established in the 1990s [35], as a molecular mechanism to describe the calcium-dependent inactivation first observed more than twenty years earlier in single-celled protozoans [11], [13] and invertebrate tissues, like snail neurones [36] and arthropod muscle [37] in the 1970s to 1980s, while more extensively elaborated over the next 30^+^ years in vertebrate preparations [6].

### Evolution of the Regulation of a Cav1.2 L-type Channel β subunit/CaM Complex in Single-celled Animals

Roger Eckert and colleagues illustrate that Ca^2+^ influx through calcium channels in single-cell *Paramecium* regulates the reversal of the orientation of cilia to swim in reverse [11]–[13]. Features of calcium channels in voltage-clamp recordings of these single-celled animals reflect a mechanism that is conserved with those in mammalian cells. *Paramecium* calcium currents show a rapid inactivation when calcium is the charge carrier that is distinguishable from a slower voltage-dependent inactivation when calcium is replaced by barium ions [11]. The degree of inactivation has a bell-shaped relation to the prepulse potential, being maximal at potentials that produce maximal Ca^2+^ entry [11], [13]. Furthermore, intracellular calcium levels dictate the kinetics of inactivation, as the calcium-dependent inactivation in *Paramecium* was calcium buffer (EGTA) sensitive [12], [13]. Single celled protozoans, including *Paramecium* have a single L-type calcium channel homolog with an extended C-terminus resembling an IQ motif (see **Figure S1**) that is associated with calmodulin itself [38] or a calmodulin-like EF-hand containing protein [39].

### The Varying Sensitivities of N- and C-lobes of CaM for Calcium Channels

CaM pre-bound to intracellular tails of calcium channels is a highly sensitive sensor for changes in calcium concentrations, through its differential occupancy of its calcium binding EF hand pairs located in each of its globular domains, with sensing of “local” calcium rise as a result of calcium passage through each channel with its high affinity C-terminal domain and the more “global” rises of calcium in the cell with its low affinity N-terminal domain [10]. Strong calcium buffers (10 mM EGTA or BAPTA) are dialyzed in cells for stable patch clamp recording and often used to inhibit calcium-dependent chloride currents in the heterologous expression of calcium channels in *Xenopus* oocytes [40], [41]. Strong calcium buffering does not disturb the high affinity, C-terminal dominant, CaM binding to Cav1 (L-type) channels, but conceals a low affinity, N-terminal dominant CaM binding to members of the non-L-type, high voltage-activated calcium channel class, Cav2 channels [25]. Structural differences likely underlie the buffering sensitivity differences of CaM binding to Cav1 and Cav2 channels, as Cav1 channels associate in reported parallel binding orientation to C-terminal end of the IQ domain [8], [9], and Cav2 channels bind farther towards the N-terminal end of the IQ domain in an anti-parallel binding orientation [42].

### Conservation of NSCaTE in Bilateral Animals

Recently, a CaM binding, N-terminal motif of L-type channels dubbed NSCaTE, or **N**-terminal **S**patial **Ca** ^2+^ **T**ransforming **E**lement was described by David Yue and collaborators in 2008 [14]. The NSCaTE motif was so named because the introduction of NSCaTE from Cav1.2 and Cav1.3 channels, into Cav2 channels, transforms Cav2 channels into ones resembling Cav1 channels in having a high buffer resistant, calcium-dependent inactivation [14]. We mined available animal genomes for the conservation of an N-terminal NSCaTE motif of L-type calcium channels outside of vertebrate Cav1.2 and Cav1.3 channels. We find a ubiquity of the NSCaTE motif with an: x-W-xxx-(I or L)-xxxx (where x are residues with propensity to form a helix, often alanines) in all animal phyla of bilaterial animals, the protostomes (nematodes, arthropods, mollusks, annelids) and deuterostomes. NSCaTE is an optional motif, and is lacking in non-mosquito insects, such as *Drosophila* and some other arthropod species, and is lacking also in some vertebrate L-type isoforms, Cav1.1 and Cav1.4 channels.

### NSCaTE Expression is Likely Regulated by Post-transcriptional Mechanism

We observe a pervasiveness of a methionine downstream from NSCaTE, which suggest that most L-type channels containing NSCaTE could generate alternative N-termini, lacking NSCaTE, whose frequency would be influenced by the variation in Kozak sequences of the Met_1_ and Met_2_ translational start sites. Interestingly, the putative translational start site, Met_2_ is typically very close (0 to 10 amino acids) from the upstream NSCaTE sequence. Alternative splicing is a common feature of the mammalian Ca_v_1.2 gene, with at least 20 of the 56 known exons which are subject to alternative splicing [43]. There are already examples in Ca_v_1.2 channels with alternative translational start sites. Mammalian Ca_v_1.2 channels have alternative exons for the first exon, dubbed exon 1a, 1b or 1c, which generates novel N-termini of 46, 17 and 10 amino acids respectively [44]–[46]. An intron splice site with an alternative first exon is uniquely featured in mammalian Ca_v_1.2 and Ca_v_1.3 channels (**Figure S2**). The alternative first exon generates tissue specific expression patterns and changes biophysical properties of mammalian Ca_v_1.2 channels [43]–[46]. NSCaTE and a putative downstream alternative start site Met_2_ is usually contained in the second exon, downstream of variable exon 1a,b,c in Ca_v_1.2 channels (**Figure S2**), and could potentially serve as a secondary translational start site in addition to the three upstream ones for mammalian channels. Calcium channels are also proteolytically cleaved at the carboxyl terminus of Ca_v_1.2 [47], [48] and Ca_v_1.3 [49] channels. The cleaved C-terminal product in Ca_v_1.2 channels serves as a transcription factor regulating gene transcription [50]. Both alternative start sites or post-translational proteolytic cleavage are possible post-transcriptional regulators of NSCaTE expression, while we can rule out transcriptional regulation as a mechanism because we observed no significant change in mRNA transcripts of LCa_v_1 channels differentially expressed from Met_1_ or Met_2_ start sites in whole snails or individual tissues (**Figure 3**).

### NSCaTE as a Quintessential Short Linear Motif (SLiM)

NSCaTE fits the profile of short linear motifs (SLiMs) which participates in protein-protein interactions and are usually associated with terminal ends of proteins at a distinct position from other functional elements [51]–[53]. SLiMs consist of an average of six amino acids (range up to 11 amino acids) [51]–[53], with a few “hotspot” residues and these, like the conserved **W** and fourth residue downstream **I**/**L** of NSCaTE, confers the affinity, specificity and free energy of CaM binding. SLiMs are often necessarily required to be near terminal ends of proteins, but variably positioned in termini elements [51]–[53], such as the 31 to 97 amino acid range that NSCaTE can extend from the start of the transmembrane region, Domain I, segment 1. SLiMs can arise independently as a result of their small size and sequence flexibility elements [51]–[53], leading to a possible separate evolution of NSCaTE in different phyla. In particular, nematodes and mosquitoes may have evolved an NSCaTE region independently, based on the clustering of their sequence variation, divergence of position of NSCaTE within the N-terminus, and presence of an additional intron in nematodes. Conserved flanking residues surrounding NSCaTE could provide fine tuning the affinity and specificity of the connectivity to interacting proteins, such as the conserved doublet of “**LS”** just upstream of non-chordate NSCaTE sequences **LS**xx**W** xxx**L**xxx (outside of nematodes), and the omnipresence of positive charge **R/K** in chordates downstream of their NSCaTE **W** xxx**L**xxx**R/K**. Short snail and mammalian NSCaTE peptides have low µM affinities that are typical for SLiMs which have a tendency to undergo transient and reversible protein interactions [51]–[53]. All N-terminal sequences containing NSCaTE are within highly disordered, unstructured sequences, but, as is typical of SLiMs, can adopt an alpha helix upon binding to their cognate target, in this case, CaM [51]–[53].

### NSCaTE Regulates Calcium-dependent Inactivation of Cav1 Channels

NSCaTE sequences in snail LCav1 and mammalian Cav1.2 and Cav1.3 increase the decay rate of calcium-dependent inactivation. The rate of calcium-dependent inactivation was significantly faster for NSCaTE containing snail *LCav1-Met1* channels revealed in the presence of low (0.5 mM EGTA buffering) conditions of HEK-293T cells. Deletion of the N-terminus of Cav1.2 (the NSCaTE containing N-terminus of Cav1.2 slows calcium-dependent inactivation) [54], [55], and similarly slows inactivation when NSCaTE binding was disrupted with W, I and R alanine replacements in the S**W**QAA**I**DAA**R** NSCaTE sequence of Cav1.2 channels expressed in a low buffering environment of *Xenopus* occytes [32].

### Current Model Suggests that Rises in Intracellular Calcium Brings NSCaTE to CaM Pre-bound to the IQ Motif

Snail NSCaTE, just like mammalian NSCaTE, [30] binds to Ca^2+^-CaM in a 2∶1 stoichiometry, which is consistent with the possible simultaneous binding of NSCaTE to both the N- and C-lobes of CaM in the absence of the IQ motif. CaM can normally pre-associate with the IQ motif at resting calcium concentrations [56], [57], and both snail and mammalian NSCaTE are able to associate with CaM even though CaM is prebound to a much higher affinity IQ motif. The calcium dependent binding of the N-terminus containing NSCaTE has been reported by others [14], [30]–[32], [54], [55]. Greater rises in intracellular calcium, would result in faster inactivation, with the participation of the N-terminal NSCaTE associating with the buffer sensitive, low affinity N-terminal CaM lobes [14]. NSCaTE is an optional motif for invertebrate L-type channels and mammalian Cav1.2 and Cav1.3 channels that increases the rate of channel refractoriness in response to excessive calcium influx, among other undefined functions that NSCaTE may have.

### Loss and Gain of Functions Associated with Vertebrate Evolution of Calcium Regulatory Feedback of Cav1 Channels

The Cav1.2 channel homolog with CaM-like binding to NSCaTE/IQ motifs in an invertebrate ancestor was retained in both vertebrate Cav1.2 and Cav1.3 channels where they have shared functions in nervous systems, heart and endocrine glands [6]. NSCaTE is notably absent in the evolution of vertebrate Cav1.1 and Cav1.4 channels, where there is little requirement for calcium-dependent inactivation. Cav1.1 serves as a mostly non-conducting and specialized voltage-sensor for ryanodine receptor coupling in vertebrate muscle triads [58], while Cav1.4 channels specialize in vertebrate T-cell homeostasis [59] and retinal phototransduction [58], [60] and possess little inactivation at all. The vertebrate brain and retina evolved specialized CaM-like calcium binding proteins, like CaBP1 [61], [62], which endow Cav1.2 channels with positive feedback, calcium-dependent facilitation, replacing CaM-dependent inactivation of Cav1 channels [55]. CaBP1 is architechturally and functionally similar to CaM and has overlapping binding sites in the C-terminal IQ motif with CaM [63] and also binds to the N-terminus, at a site that is downstream from the CaM binding NSCaTE motif [33].

### NSCaTE Evolved in Parallel with the Evolution of the Heart

Cav1.2 channel homologs evolved with multiple and tight controls on excessive calcium influx with pre-bound subunits, like β subunits, that alters voltage-sensitive properties and a C-terminal bound CaM-like protein to monitor intracellular calcium levels. The regulation of Cav1.2/β subunit/CaM-like complex is conserved in the L-type calcium channels underlying the ciliary reversal in single cell protozoans as it is in complex tissues of mammalian brain and heart. A secondary and optional CaM binding motif, NSCaTE first evolves later in multi-cellular, bilateral organisms with a body cavity (coelom) containing the first internal organs, such as the nutritive, primitive heart (nematode pharynx) [64], [65] and endocrine glands (neurosecretory cells). Nervous systems and contractile muscle fibers predate bilateral organisms in cnidarians and flatworms. It is attractive to consider that NSCaTE was an adaptation associated with the first rhythmically contracting primitive heart, as a preventative measure against arrhythmias associated with excessive calcium influx and prolonged action potentials.

## Materials and Methods

### Data-mining and Computational Analyses of L-type Orthologs

N-termini and C-termini sequences were gathered for orthologs of L-type channels by BLAST data-mining of available genomic databases such as NCBI (Bethesda, MD), Joint Genome Institute, Department of Energy and University of California (DOE-JGI), Washington University in St. Louis (Genome Institute at WUSTL), Baylor College (HGSC), Broad Institute of MIT and Harvard. Multiple alignments of amino acid sequences and gene tree making were conducted using Phylogeny.fr [66]. Running window of amino acid similarity of multiple aligned sequences was conducted using Plotcon within EMBOSS [67].

### Cloning and HEK293T Cell Transfection of Snail LCav1 Channel N-terminal Variant

PCR was used to create LCa_V_1 channels with an alternative start site 112 amino acids downstream of the long form of the channel, removing a predicted NSCaTE sequence at positions (90 to 101) in the existing full-length LCa_V_1 (Genbank: AF484079, 2,078 aa) cloned in mammalian expression vector, pIRES2-EGFP plasmid [20], [21]. pIRES2 is a bicystronic vector which generates a post-translationally cleaved EGFP reporter for easy detection of LCav1 expressing channels using green fluorescence on an epifluorescent microscope. Plasmid sequences were verified by sequencing (TCAG, Sick Kids Hospital, Toronto, ON). LCav1 channels were transient transfected along with required α_2_δ_1_ and either β1b or β2a subunits in HEK293T cells using calcium phosphate precipitation. The method of plasmid transfection and whole cell patch clamp recording of calcium currents were carried out as illustrated in a video journal (JoVE) [68] and other publications [20], [22], [24], [68], [69]. Plasmids for rat α2δ1 (NM_012919, 1,091 aa), rat β2a (NP_446303, 604 aa) and rat β1b (NM_017346, 597 aa) were a generous gift from Terry Snutch (Univ. British Columbia) via Gerald Zamponi (Univ. of Calgary).

### Quantitative PCR of Transcripts Containing or not Containing NSCaTE

Quantitative reverse-transcription PCR (qPCR) of snail tissues and whole animals (embryos, juveniles and adults) has been introduced in other manuscripts [15], [70], [71]. mRNA was extracted from 100% embryos identified by morphological features of animals within egg capsules [72], and the shell lengths (1–1.5 cm and 2–2.5 cm respectively) of juvenile and adult snails respectively [73]. *Lymnaea* Ca_v_1 transcripts were amplified by quantitative RT-PCR (qPCR) with a forward and reverse primer pair designed against LCa_v_1 to amplify a 138 bp sequence just upstream of the NSCaTE sequence, respectively (LCav1-Met1f: 5′ GAGGTAGAGGAAGGAGGAGGAG 3′ and LCav1-Met1b: 5′ TGCCAGAGTCTGTTGTATTCAGAG 3′), and a primer pair designed to amplify a 145 bp sequence just downstream of NSCaTE: (LCav1-Met2f: 5′ TACAGGTTGCAGAATACAAAGCAT 3′ and LCav1-Met2b: 5′ AAGACATATTCAATCCGATCCAGT 3′). Quadruplicate qPCR cycle threshold (CT) values for each amplicon were averaged and normalized against averaged CT data for control gene HPRT1 using the ratio [74]: (E_target gene_)^ΔCTtarget gene^/(E_HPRT1_)^ΔCTHPRT1^. Amplification efficiencies (E values) for each primer pair was determined by generating standard curves using 1∶5 serial dilutions of pooled cDNA from all RNA extracts as template.

### Electrophysiology

Electrophysiological recordings were carried out at room temperature with an Axopatch 200B or Multiclamp 700B amplifier (Axon Instruments, Union City, CA) through a PC computer equipped with a Digidata 1440A analog-to-digital converter in conjunction with pClamp10.1 software (Molecular Devices, Sunnyvale, California). Cells were bathed in external solution containing barium (10 mM BaCl2) or calcium (10 mM CaCl2) as the charge carrier and 1 mM MgCl2, 10 mM HEPES, 40 mM TEA-Cl, 80 mM CsCl, 10 mM Glucose, adjust pH to 7.2 with TEA-OH, filter through 0.22 µm filter). Patch pipettes (World Precision Instruments, Sarasota, Florida were filled with internal solution (106 mM Cs-methanesulfonate, 4 mM MgCl2, 9 mM EGTA, 9 mM HEPES, 2 mM MgATP adjust pH to 7.2 with CsOH, filter through 0.22 µm filter) and had resistances of 2–5 MΩ,. For the low EGTA experiments, the internal solution was 114.5 mM Cs-methanesulfonate, 4 mM MgCl2, 0.5 mM EGTA, 9 mM HEPES, 2 mM MgATP adjust pH to 7.2 with CsOH Recorded currents were digitized at a sampling frequency of 2 kHz and filtered at 10 kHz using a low-pass Bessel filter. Only recordings with minimal leak (<10%) were used for analysis, and offline leak subtraction was carried out using the Clampfit 10.1software (Molecular Devices, Sunnyvale, California). Series resistance was compensated to 70% (prediction and correction;10 µs lag). All values are expressed as the mean ± SEM, with statistical analyses using a one-way ANOVA.

Current-voltage relationships were obtained by holding cells at −60 mV before stepping to test potentials ranging from −50 to +60 mV for 300 ms. The activation, inactivation and recover from inactivation curves were generated and analysed as described previously [20], [68].

### CaM Preparation and Peptide Synthesis

Wild type rat CaM in pET9d (Novagen) was expressed and purified on an AKTA100 FPLC using standard protocols involving a HiScreen Phenyl Sepharose high-sub column (GE Healthcare) followed by gel-filtration with a Sephadex 75 10/300 and/or a 16/60 column to remove all contaminating proteins (verified by SDS-PAGE and mass spectrometry), then aliquoted and stored at −80°C. Synthetic peptides corresponding to mammalian NSCaTE = SWQAAIDAARQAKLMGS, *Lymnaea* NSCaTE = GWSTALAAAQGAATVRK, snail LCa_V_1 IQ = KFYATFLIQDYFRRFKKRKEQ, and mammalian Ca_v_1.2 IQ (α_1c_) = KFYATFLIQEYFRKFKKRKEQ were ordered from CanPeptide Inc (Pointe-Claire, Quebec) and were of >95% purity according to QA specifications. All peptides were resuspended in mili-Q deionized water to 1 mM stocks and stored at −80°C in small aliquots.

### Gel Shift Mobility Assays

Gel shift mobility assays were completed following the protocol outlined. Briefly, 300 pmol of WT CaM were loaded in each well. Every gel contains a Ca^2+^-CaM-only control in the first lane, and an IQ peptide-CaM control in the second lane where NSCaTE competition was performed. Peptides and CaM were pre-incubated at 4°C for 1 h in binding buffer, and gels were run on ice with jacketed water flow set to 4°C (with 0.1 mM CaCl_2_ in running/casting buffers). In all cases, native 15% separating and 4% stacking gels were used.

### Isothermal Titration Calorimetry

All ITC recordings were performed on a Microcal ITC200 from Microcal (Northampton, MA) at 25°C. In all experiments the buffer used was 25 mM Tris-Cl pH 7.5, 75 mM NaCl and 0.5 mM CaCl_2_ and was identical between cell and syringe. Buffer into buffer, peptide into buffer and buffer into CaM controls showed no significant baseline decay or drift. 39 µL of peptide was titrated into 200 µL of CaM at varying concentrations (optimal starting conditions were determined empirically), typically from 250 µM peptide into 25 µM CaM to 500 µM peptide into 50 µM CaM, over the course of 20–30 injections at 2–3 min intervals. Data analysis was performed using Origin ITC200 Origin70 module with pre-loaded fitting equations for one- and two-sites models. The one-set-of-sites model was found to be applicable to all experiments.

### Circular Dichroism (Spectropolarimetry)

Far-UV CD recordings were performed on the Jasco-715 spectropolarimeter (Jasco Instruments, NS) at room temperature, using a 0.1cm quartz cuvette. Settings were as follows: 250–190 nm range, 20 nm/min, 1s response time, 0.5 nm bandwidth and 100mdeg sensitivity. Each scan was an average of 16 accumulations, and all samples were baseline corrected each day (to the buffer). 0.1 mM CaCl_2_ was added to the phosphate buffer for efficient CaM-peptide binding. In all cases, 10 µM CaM was recorded with subsequent additions of peptide (1 mM stock peptide into a 250 µL cuvette total volume). TFE recordings were performed using pure 2,2,2-trifluoroethanol (Sigma Aldrich) after thorough pre-mixing and incubation with peptides in phosphate buffer (10 mM Na_2_HPO_4_, pH 7.5).

## Supporting Information

Figure S1 **Expanded multiple alignment of L-type calcium channel C-termini illustrating the high conservation of the calmodulin-binding Pre-IQ/IQ region.** The C-terminal region from the end of Domain IV to the end of the Pre-IQ/IQ is the most conserved, continuous stretch of amino acid sequence in any voltage-gated calcium channel and highly conserved in all metazoans. Calmodulin is also highly conserved (96–98% identical) among metazoans, and likely required for the ubiquitous calcium dependent inactivation observed in all L-type calcium currents from single-celled eukaryotes (*Paramecium*) (Brehm *et al.*, 1978;Brehm *et al.*, 1980) to humans (Christel *et al.*, 2012).
(TIF)

Figure S2 **Expanded multiple alignment of L-type calcium channel amino -termini illustrating the conservation of NSCATE in coelomate animals (nematodes, arthropods, mollusks, annelids, echinoderms, chordates).** NSCATE is a highly conserved, Wxxx(I/L)xxx short linear motif that forms a predicted helix in solution, usually associated with a methionine just downstream from NSCATE (0 to 10 amino acids in non-nematodes). NSCATE and a downstream methionine residue, are the only consistent features in the amino terminus of L-type channels between coelomate protostomes and vertebrate Ca_v_1.2 and Ca_v_1.3 channels, which otherwise is highly variable, mostly unstructured sequence, varying in length and amino acid sequence identity. Red lines are the locations of known introns. The intron that separates the amino-terminus and the first transmembrane segment (DI, S1), is often an unusual AT-AC splice site, and is shared between Na_v_ and Ca_v_ channels.
(TIF)

Figure S3 **Prediction of disorder/unstructured regions and secondary structure (α-helix, β-strand, coils) of the amino-terminus of deuterostome and annelid L-type Ca_v_1 channels.** Consensus NSCATE sequence (contained in boxed amino acids): Wxxx(I or L)xxxx where x (blue amino acids) form a predicted helix. NSCATE is conserved in a predicted highly disordered, unstructured region (red stars) of L-type channels. NSCATE has properties of a typical Short Linear Motif (SLiM), which resides within disordered region. Typically SLiMs form a structured secondary structure (helix) induced by protein interaction (in this case with Ca^2+^-CAM). A typical SLiM is ∼6 contiguous amino acids, but can range from 3 to 11 amino acids long, with critical hotspot residues like the conserved W and (I or L), that form the majority of the free energy of binding and determine most of the affinity and specificity of Ca^2+^-CAM binding. Disorder and secondary structure predictions were made with DISOPRED2 and PSIPRED3.3 (UCL-CS Bioinformatics).
(TIF)

Figure S4 **Prediction of disorder/unstructured regions and secondary structure (α-helix, β-strand, coils) of the amino-terminus of mollusks, arthropods and nematode L-type Ca_v_1 channels.** Consensus NSCATE sequence (contained in boxed amino acids): xWxxx(I or L)xxxx where x (blue amino acids) form a predicted helix. NSCATE is conserved in a predicted highly disordered, unstructured region (red stars) of L-type channels. Disorder and secondary structure predictions were made with DISOPRED2 and PSIPRED3.3 (UCL-CS Bioinformatics).
(TIF)

Table S1 **Expanded table of L-type channels in the animal kingdom and the conservation of calmodulin binding, N-terminal NSCATE and C-terminal Pre-IQ/IQ motifs.** A C-terminal IQ motif is ubiquitously featured in known L-type channels outside of possibly the more basal animals, such as single-celled coanoflagellates. Calmodulin and Ca_v_β subunits were likely pre-associated to the earliest eukaryotic L-type channels since both accessory subunits are featured in all known genomes where L-type channels are found. While the C-terminal IQ motif is ubiquitously found in L-type channels, NSCATE is an optional short linear motif in the N-terminus of L-type channels. NSCATE has a common motif: xWxxx(I/L)xxx, and a predicted helix structure in solution, sandwiched in a highly variable and disordered N-terminus, at a variable distance (36 to 97 aa) from the first membrane segment (Domain I, Segment 1). NSCATE first appears in coelomate animals, where it is usually present, except for some arthropod species. In most L-type channel with an NSCATE motif, there is a methionine just downstream from NSCATE (0 to 10 amino acids in non-nematodes), which creates possible alternative translational start sites to generate L-type channel proteins with and without NSCATE. Calmodulin-like, Calcium Binding Protein 1, CaBP1 also binds to the N- and C-termini of L-type channels, but this was an add-on that appeared in vertebrates only, ∼100s of millions of years after the evolution of L-type channels pre-associated with calmodulin and Ca_v_β subunits in early eukaryotes.
(TIF)
