# Correction: The Calmodulin-Binding, Short Linear Motif, NSCaTE Is Conserved in L-Type Channel Ancestors of Vertebrate Cav1.2 and Cav1.3 Channels

**DOI:** 10.1371/annotation/7fc1cd8d-1eeb-4ab7-929b-96529cab1c1f

**Published:** 2014-01-13

**Authors:** Valentina Taiakina, Adrienne N. Boone, Julia Fux, Adriano Senatore, Danielle Weber-Adrian, J. Guy Guillemette, J. David Spafford

In Figure 6, Panel A is missing text. The correct version of Figure 6 can be viewed here: http://plosone.org/corrections/pone.0061765.g006.cn.tif

