# Supplementary figures and images for: The Calmodulin-Binding, Short Linear Motif, NSCaTE Is Conserved in L-Type Channel Ancestors of Vertebrate Cav1.2 and Cav1.3 Channels
Source: PLoS One. 2013 Apr 23;8(4):e61765. doi: 10.1371/journal.pone.0061765 (PMC3634016; doi:10.1371/journal.pone.0061765)

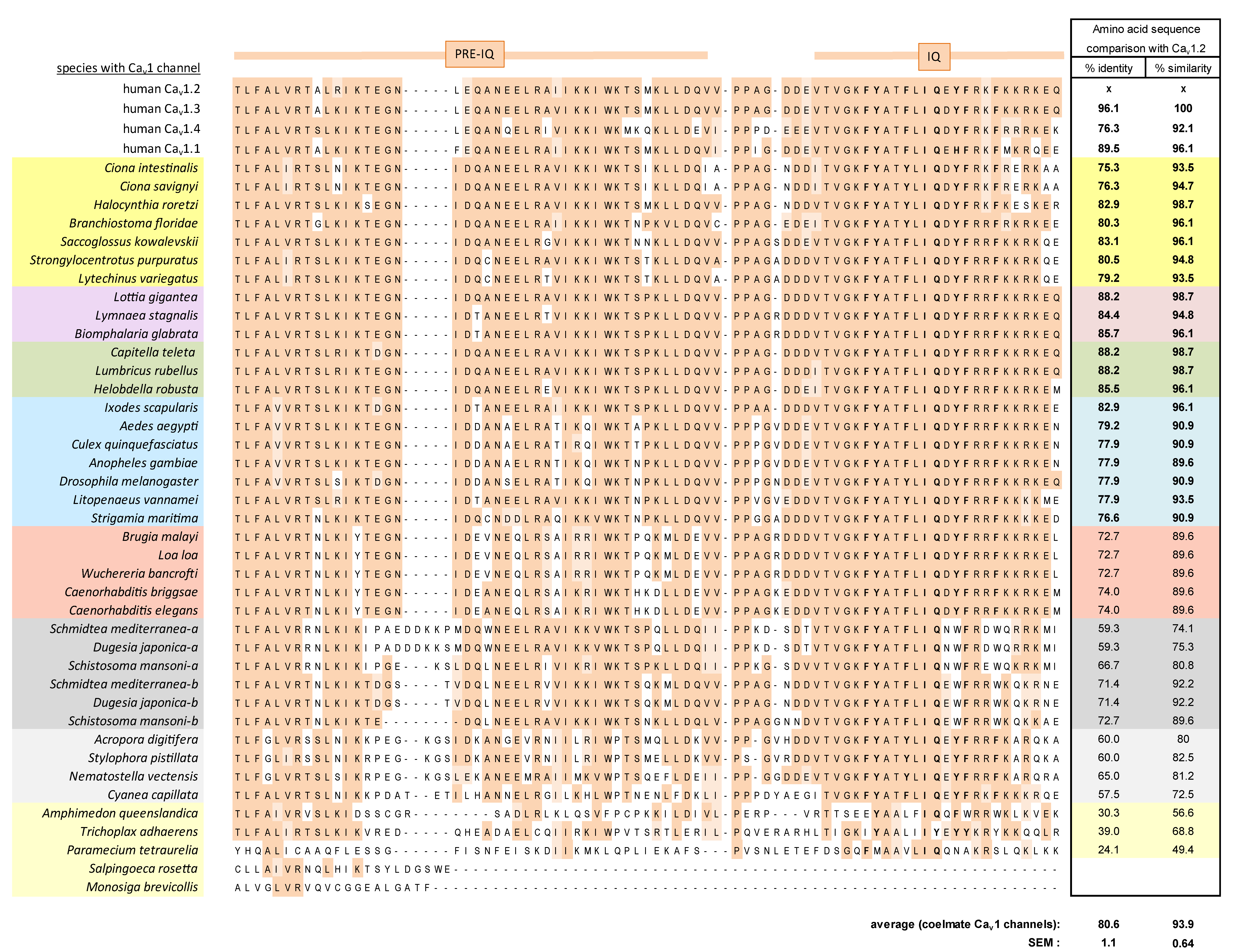

Supplement: Figure S1 — Expanded multiple alignment of L-type calcium channel C-termini illustrating the high conservation of the calmodulin-binding Pre-IQ/IQ region. The C-terminal region from the end of Domain IV to the end of the Pre-IQ/IQ is the most conserved, continuous stretch of amino acid sequence in any voltage-gated calcium channel and highly conserved in all metazoans. Calmodulin is also highly conserved (96–98% identical) among metazoans, and likely required for the ubiquitous calcium dependent inactivation observed in all L-type calcium currents from single-celled eukaryotes (Paramecium) (Brehm et al., 1978;Brehm et al., 1980) to humans (Christel et al., 2012). (TIF) [file pone.0061765.s001.tif]

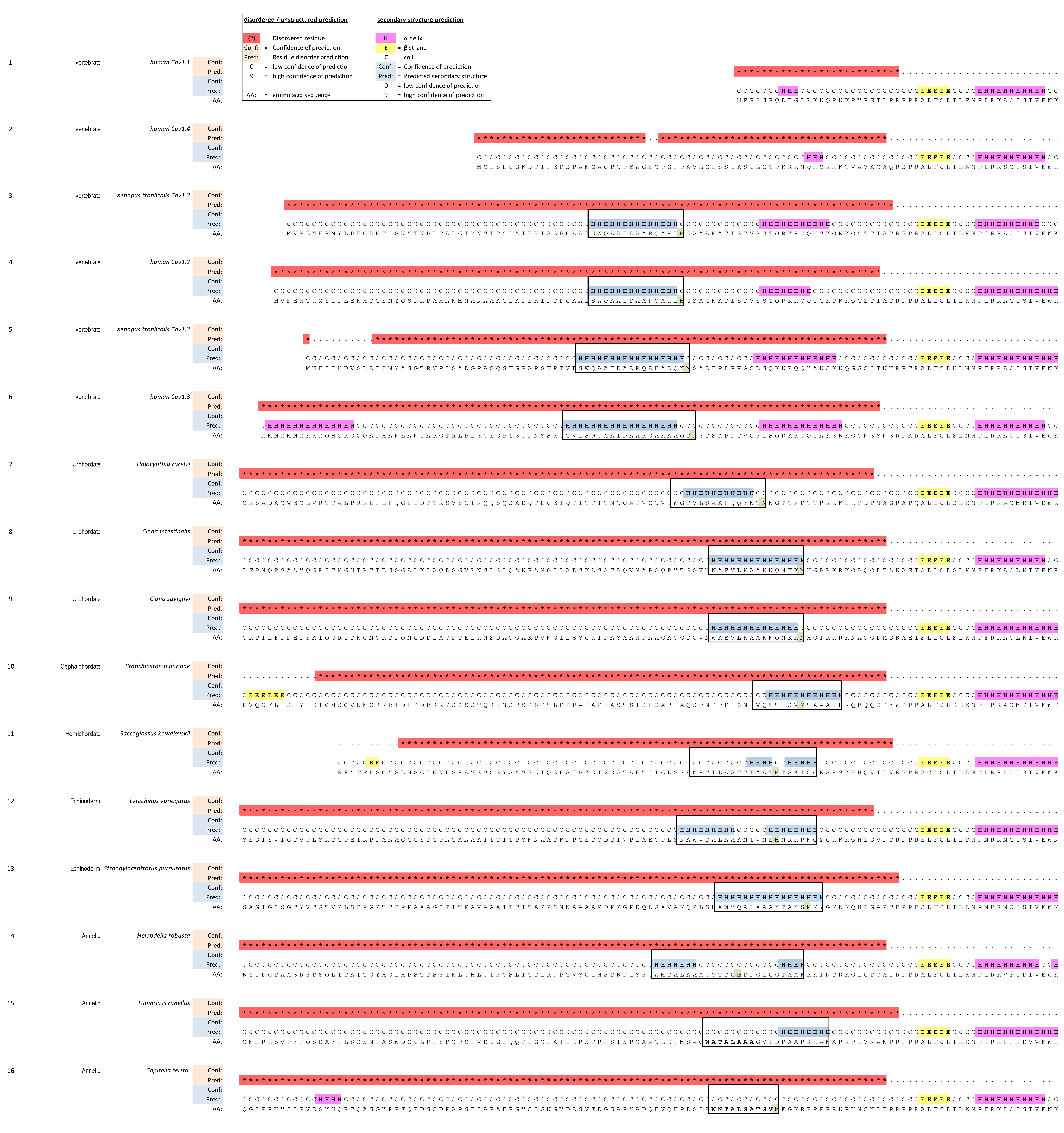

Supplement: Figure S3 — Prediction of disorder/unstructured regions and secondary structure (α-helix, β-strand, coils) of the amino-terminus of deuterostome and annelid L-type Cav1 channels. Consensus NSCATE sequence (contained in boxed amino acids): Wxxx(I or L)xxxx where x (blue amino acids) form a predicted helix. NSCATE is conserved in a predicted highly disordered, unstructured region (red stars) of L-type channels. NSCATE has properties of a typical Short Linear Motif (SLiM), which resides within disordered region. Typically SLiMs form a structured secondary structure (helix) induced by protein interaction (in this case with Ca2+-CAM). A typical SLiM is ∼6 contiguous amino acids, but can range from 3 to 11 amino acids long, with critical hotspot residues like the conserved W and (I or L), that form the majority of the free energy of binding and determine most of the affinity and specificity of Ca2+-CAM binding. Disorder and secondary structure predictions were made with DISOPRED2 and PSIPRED3.3 (UCL-CS Bioinformatics). (TIF) [file pone.0061765.s003.tif]

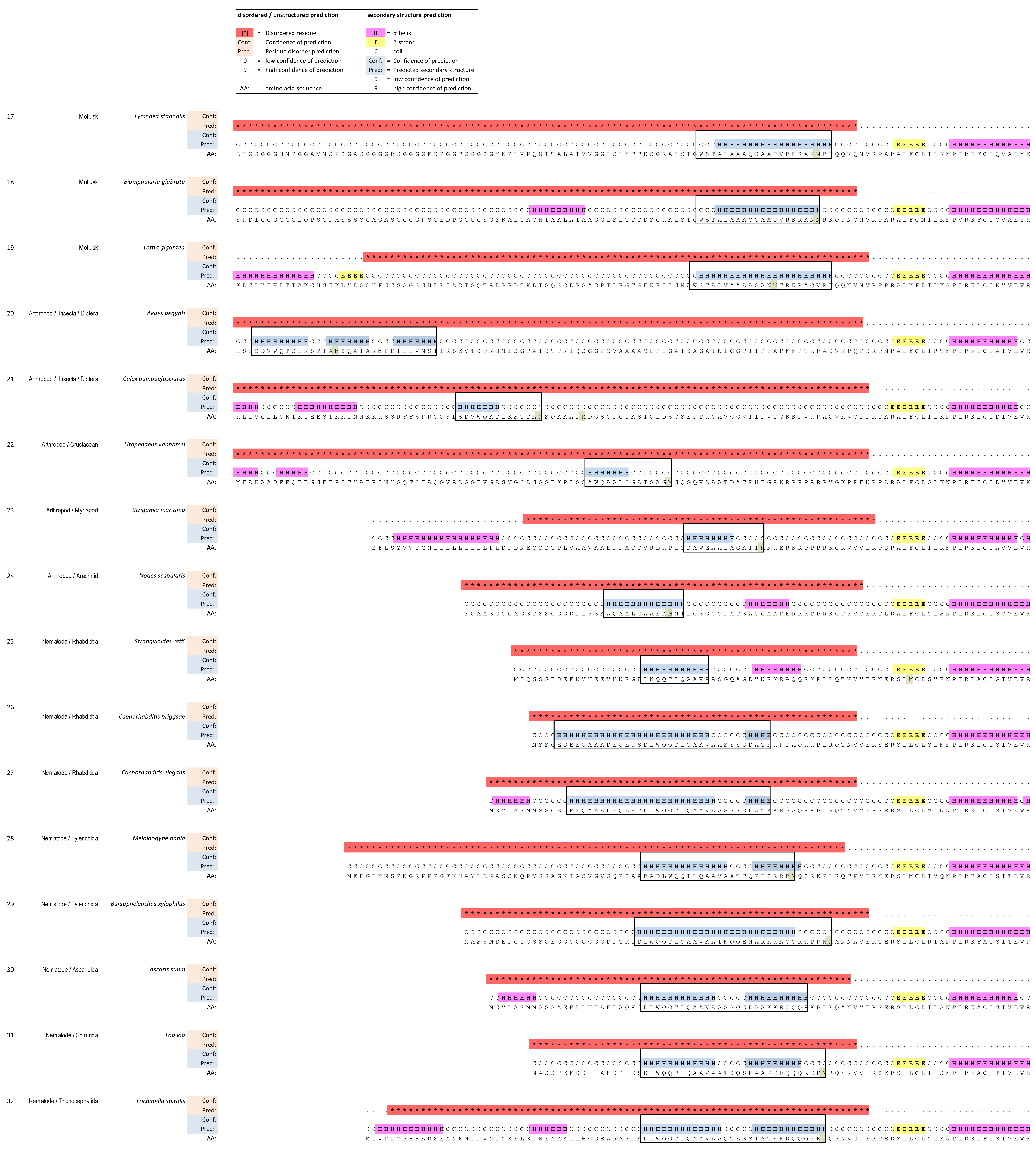

Supplement: Figure S4 — Prediction of disorder/unstructured regions and secondary structure (α-helix, β-strand, coils) of the amino-terminus of mollusks, arthropods and nematode L-type Cav1 channels. Consensus NSCATE sequence (contained in boxed amino acids): xWxxx(I or L)xxxx where x (blue amino acids) form a predicted helix. NSCATE is conserved in a predicted highly disordered, unstructured region (red stars) of L-type channels. Disorder and secondary structure predictions were made with DISOPRED2 and PSIPRED3.3 (UCL-CS Bioinformatics). (TIF) [file pone.0061765.s004.tif]
